# UV-C Irradiation of Rolled Fillets of Ham Inoculated with *Yersinia enterocolitica* and *Brochothrix thermosphacta*

**DOI:** 10.3390/foods9050552

**Published:** 2020-05-01

**Authors:** Julia Reichel, Corinna Kehrenberg, Carsten Krischek

**Affiliations:** 1 Institute for Food Quality and Food Safety, Foundation University of Veterinary Medicine Hannover, Bischofsholer Damm 15, 30173 Hannover, Germany; julia.reichel@tiho-hannover.de; 2 Institute for Veterinary Food Science, Justus-Liebig-University Giessen, Frankfurter Str. 92, 35392 Giessen, Germany; corinna.kehrenberg@vetmed.uni-giessen.de

**Keywords:** ham, ultraviolet irradiation, photoreactivation, *Yersinia enterocolitica*, *Brochothrix thermosphacta*

## Abstract

Bacteria on ready-to-eat meat may cause diseases and lead to faster deterioration of the product. In this study, ready-to-eat sliced ham samples were inoculated with *Yersinia enterocolitica* or *Brochothrix thermosphacta* and treated with ultraviolet (UV) light. The initial effect of a UV-C irradiation was investigated with doses of 408, 2040, 4080, and 6120 mJ/cm^2^ and the effect after 0, 7, and 14 days of refrigerated storage with doses of 408 and 4080 mJ/cm^2^. Furthermore, inoculated ham samples were stored under light and dark conditions after the UV-C treatment to investigate the effect of photoreactivation. To assess the ham quality the parameters color and antioxidant capacity were analyzed during storage. UV-C light reduced *Yersinia enterocolitica* and *Brochothrix thermosphacta* counts by up to 1.11 log_10_ and 0.79 log_10_ colony forming units/g, respectively, during storage. No photoreactivation of the bacteria was observed. Furthermore, significantly lower a* and higher b* values after 7 and 14 days of storage and a significantly higher antioxidant capacity on day 0 after treatment with 4080 mJ/cm^2^ were detected. However, there were no other significant differences between treated and untreated samples. Hence, a UV-C treatment can reduce microbial surface contamination of ready-to-eat sliced ham without causing considerable quality changes.

## 1. Introduction

The consumption of ready-to-eat (RTE) products has risen in recent years due to their easy availability and good quality [1]. In this category meat products like rolled fillets of ham are frequently consumed. However, as during production, slicing and further handling the ham can be contaminated with bacteria, present in the food processing plant and household, and as the product is consumed without previous heating, RTE products might pose a risk for food-borne illnesses.

It is known that contaminated pig carcasses can transmit pathogens such as *Yersinia* (*Y*.) *enterocolitica* or spoilage bacteria such as *Brochothrix* (*B*.) *thermosphacta* from the slaughterhouse to meat processing plants, where they in turn can contaminate surfaces and equipment, e.g., slicing machines [2,3]. Thus, even hygienically safe meat products can be re-contaminated with these bacteria, e.g., during slicing. *Y. enterocolitica*, which were found in RTE pork products, can cause human yersiniosis, a food-borne bacterial disease, often associated with diarrhea and bowel inflammation [4]. Cases of yersiniosis are still high in some countries such as Germany. In the European Union a total of 6806 cases of yersiniosis were reported in 2018 [5]. Pathogenic *Y. enterocolitica* are most frequently found in pigs and therefore, pig meat products may pose a risk to the consumer. The highest bacterial counts of *Y. enterocolitica* can be determined in the feces and tonsils of pigs and thus, in addition to fecal contamination, the handling of the head during slaughter may lead to a spreading of the microorganism [6,7]. *B. thermosphacta* is an important spoilage bacteria frequently found on meat and meat products [8]. In addition to quality changes like off-odors and discoloration of the food, these bacteria can also lead to gastrointestinal disturbances, if the product is contaminated with high *B. thermosphacta* concentrations [9]. Growth of *Y. enterocolitica* and *B. thermosphacta* is influenced by numerous intrinsic or extrinsic factors like temperature, pH value, water activity, or the specific gas composition in modified atmosphere packages (MAP). The latter is important, as ham is frequently purchased in MAP [10,11].

Although there are already many measures during production to increase the hygienic safety of the product such as disinfection of food contact surfaces, maintenance of the cold chain and packaging under modified atmosphere, methods to reduce the contamination of RTE products like ham after production are of great interest for the food industry. Treatment with ultraviolet (UV) light has long gained in importance as a decontamination technique as it requires neither chemicals nor heat. UV-C light with wavelengths between 200 and 280 nm and especially the wavelength of 254 nm has proven to be the most effective within the UV spectrum (100–400 nm), as it generates pyrimidine dimers as well as pyrimidine-pyrimidone (6-4) photoproducts and denatures microbial DNA. These alterations may be lethal to bacteria or may reduce their reproduction abilities [12]. However, photoreactivation is an efficient repair mechanism of bacteria to (partly) recover their viability and to minimize the effects of the UV treatment. The photolyase, an important enzyme of the photoreactivation process, is activated by visible light and catalyzes the repair of the damaged DNA without excising parts of the DNA strand [13].

Inactivation studies using UV-C irradiation have already demonstrated effectiveness in lowering bacterial counts on meat without causing quality changes [14,15]. However, the effect is limited to surface decontamination, as UV-C light cannot penetrate deeper layers of the tissue [16]. As the chemical composition of the product has a considerable influence on the effectiveness of the UV-C treatment [17], the question arises, how this preservation method reduces the bacterial contamination of processed meat products such as rolled fillets of ham. Beside this, it is important to know how UV-C irradiation might not only influence bacterial numbers, but also other quality parameters of the meat products such as color values or antioxidant activities. Therefore, the aim of the present study was to analyze the effectiveness of UV-C treatment on *Y. enterocolitica* and *B. thermosphacta* counts and product quality parameters of rolled fillets of ham. *Y. enterocolitica* was chosen in the present study, as, considering the above described information, higher risks for contamination of pork ham during production and packaging and therefore of infections of humans could be assumed in comparison to other important pathogens like *Listeria monocytogenes* or *Staphyloccocus aureus*. Even though *Y. enterocolitica* does not find conditions for growth in some pork products such as ham, the inactivation of the pathogen on meat and meat products plays an important role and some approaches with this aim have already been published [18]. Therefore, the focus of this study is on reducing bacterial contamination with the UV-C treatment and not on the influence of bacterial growth.

## 2. Materials and Methods

### 2.1. Ethics Statement

In the study, pig muscles were collected from a commercial pig slaughterhouse, UV-C treated and analyzed after processing to rolled fillets of ham. The slaughterhouse considered all European and German animal welfare regulations for transport, handling, and slaughter of the animals.

### 2.2. Test Organisms

The *Y. enterocolitica* isolate used for this study was obtained from the tonsils of a wild boar and was part of the strain collection of the Institute for Food Quality and Food Safety, Foundation University of Veterinary Medicine Hannover, Germany. The *B. thermosphacta* strain DSM 20171 (isolated from fresh pork sausage) was obtained from the German collection of microorganisms and cell cultures (DSMZ, Braunschweig, Germany). The isolates were stored at −80 °C in cryotubes until use. Both bacterial strains were used based on previous in vitro experiments and their porcine origin.

### 2.3. UV Equipment

The irradiation process was performed with the UV-Cabinet-H-NX-1/5 (Light Progress S.r.l., Anghiari, Italy). It has five 40 W low pressure mercury UV lamps which emit light with a wavelength of 253.7 nm. The UV-C intensity was determined with the Handheld HI 1 and the UV-Sensor SI 1 (UV-technik Meyer GmbH, Ortenberg, Germany). The UV-C intensity was 6.8 mW/cm^2^. As the UV-C dose (in mJ/cm^2^) is the product of the UV-C intensity (in mW/cm^2^) and the exposure time (in s), it was changed by altering the exposure time.

For each experiment the lamps were switched on at least 10 min before exposure, as previous experiments showed that this period was sufficient to guarantee a constant UV intensity output. The samples were irradiated at room temperature (ca. 22 °C).

### 2.4. Overview of the Experiments

**Figure 1:**
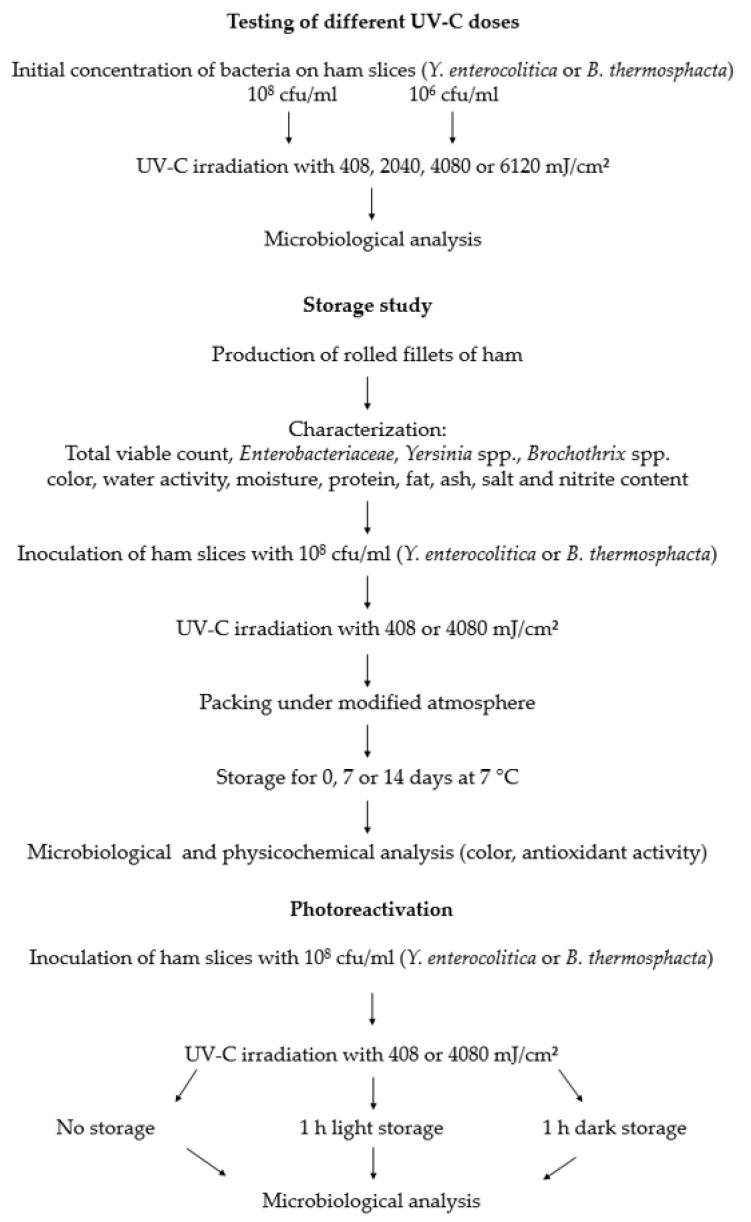
Overview of the experiments performed in the present study.

Figure 1 gives an overview of the experiments. First, various UV-C doses were tested for their ability to reduce the bacterial count on rolled fillets of ham. In addition, different initial concentrations of bacteria were used to analyze in which way the reduction of bacteria was influenced by the amount of bacteria on the product.

For the storage study the ham was inoculated with either *Y. enterocolitica*, or *B. thermosphacta*, UV-C irradiated (408 or 4080 mJ/cm^2^) and packed under modified atmosphere. On days 0, 7, and 14 samples for microbiological and physicochemical (color and antioxidant activity) investigations were removed and analyzed.

In additional experiments the influence of photoreactivation after the UV-C irradiation of ham was tested.

### 2.5. Material

For the analysis of the dose dependent bacterial reduction, slices of rolled fillets of ham of the type of an air dried ham from five different batches of the same brand were purchased from a local supermarket. Additionally, three different batches of the ham were purchased to analyze whether photoreactivation occurs. Each package contained 100 g ham slices, packaged in polypropylene under modified atmosphere. The sales designation is low fat (maximum 2% fat) cured rolled fillet of ham which is produced from the *Musculus longissimus thoracis et lumborum* (LTL). The package was mostly transparent and partly painted. The ham contained the following ingredients: pork, iodized salt, potassium iodate, dried glucose syrup, dextrose, sugar, sodium acetate, sodium citrate, sodium ascorbate, sodium nitrite, spice extracts, beech wood smoke.

For the storage study, LTL from the left side of three different female pigs were collected from a local slaughterhouse 24 h after slaughter. Firstly, the LTL was freed from fat and tendons. For the dry curing process, the meat was evenly rubbed with 50 g nitrite curing salt (CS) per kg meat (meat: CS ratio: 20:1), vacuum packed, and stored at 4 °C. The CS consists of 99.5% sodium chloride (NaCl) and 0.5% sodium nitrite (NaNO_2_). After 14 days, the meat was unpacked and hung at 4 °C for a further 4 weeks. After this storage period the color of the ham, the water activity (a_w_), moisture, protein, fat, ash, NaCl, and NaNO_2_ content were analyzed. Furthermore, the total viable count (TVC) as well as the bacterial counts of *Enterobacteriaceae*, *Yersinia* spp., and *Brochothrix* spp. were determined. For further analyses the ham was portioned into slices (but not rolled) with a thickness of 1.5 mm and a mass of about 10 g.

### 2.6. Preparation and Treatment of Ham

*B. thermosphacta* was cultured on Columbia agar with sheep blood (Oxoid GmbH, Wesel, Germany) for 24 h at 25 °C and *Y. enterocolitica* was cultured on plate count agar (Oxoid) for 24 h at 30 °C. Colonies were removed from the plate and suspended in 4 mL sterile saline solution (0.85% NaCl). The bacterial suspension was adjusted to a 1.0 McFarland turbidity standard (approximately 10^8^ colony forming units (cfu)/mL) with a densimat (BioMérieux SA France IDN 013615, Craponne, France). In further experiments, the bacterial suspension was adjusted to 10^6^ cfu/mL to test if a lower initial bacterial concentration influences the reductions.

For the analysis of the dose dependent reduction and the photoreactivation the purchased ham slices, and for the storage studies the slices from the self-made hams, were evenly inoculated with 0.5 mL of the bacterial suspensions (either *B. thermosphacta*, or *Y. enterocolitica*). Twenty minutes after inoculation the slices were treated with UV-C. Therefore, the samples were placed at a distance of 10 cm under the UV lamp and either treated with doses of 408, 2040, 4080, or 6120 mJ/cm^2^ (dose dependent reduction) or with 408 or 4080 mJ/cm^2^ (storage study and photoreactivation study), respectively. The doses for the storage study were selected because in the initial reduction studies they either showed slight but not significant reductions (408 mJ/cm^2^) or clearly significant reductions (4080 mJ/cm^2^) in the bacterial counts.

For the photoreactivation study, the samples were divided into three different treatment groups. The first group was microbiologically analyzed immediately after the UV-C irradiation. The second group was exposed to visible light for 1 h after irradiation and then analyzed, whereas the third group of samples was stored without previous light exposure.

The analyses of the dose dependent reductions were performed with three (*B. thermosphacta*) or five (*Y. enterocolitica*) different batches of rolled fillets of ham, purchased from a supermarket and the analyses of the photoreactivation were performed with three different batches. The storage study was performed with three different batches of rolled fillets of ham, made from the LTL of three pigs (*n* = 3).

### 2.7. Storage

The UV-C treated and untreated (control) samples were packed under modified atmosphere (70% O_2_ and 30% N) in plastic trays (ES-Plastic GmbH, Hutthurm, Germany) and were stored at 7 °C until further analysis. On the treatment day (designated as day 0) and the days 7 and 14 samples were collected for microbiological and physicochemical analyses. The used samples were excluded from further experiments.

### 2.8. Microbiological Parameters

The samples were homogenized in bags (Stomacher 400 Strainer Bags, Seward limited, Worthing, UK) with a Stomacher (Stomacher 400 Circulator, Seward) in a dilution of 1:10 with sterile saline solution with peptone (0.85% NaCl and 0.1% peptone) for 2 min at 230 rpm. After homogenization, the samples were serially diluted. *Yersinia* spp. counts were determined using cefsulodin irgasan novobiocin agar (Oxoid) at 30 °C for 24 h (ISO 10273:2017). *Brochothrix* spp. were quantified using streptomycin-inosit-neutral-red agar plates (Oxoid) at 25 °C for 48 h according to Hechelmann [19]. *Enterobacteriaceae* were grown on violet red bile glucose agar plates (Oxoid) at 37 °C for 48 h (ISO 21528:2017) and TVC were determined on plate count agar (Oxoid) at 30 °C for 72 h (ISO 4833-1:2013).

The detection limits for TVC and *Enterobacteriaceae* were 1.0 log_10_ cfu/g and for *Y. enterocolitica* and *B. thermosphacta* 2.0 log_10_ cfu/g. If no colonies were determined on the agar plates with the initial dilution, the half detection limit (0.7 log_10_ or 1.7 log_10_ cfu/g meat) was used for further calculations.

### 2.9. Physical and Chemical Parameters

A Minolta CR 400 colorimeter (Konica-Minolta GmbH, Langenhagen, Germany; 2° standard observer, D65 illuminant, 8 mm measuring field) was used to analyze the instrumental color parameters. In advance, it was calibrated with a standard white plate (Konica-Minolta GmbH). The results are expressed as CIE L* (lightness), a* (redness), and b* (yellowness). The color measurements took place immediately after the package was opened and the ham was placed on a white background. Each value was an average of five measurements. Additionally, the total color difference delta (Δ) E was determined between the treatment groups using the following formula:

(1) $$\Delta E=\sqrt{{\left({\Delta L}^{*}\right)}^{2}+{\left({\Delta a}^{*}\right)}^{2}+{\left({\Delta b}^{*}\right)}^{2}}$$

ΔE shows an objective difference between two colors and described the difference of L*a*b*-measurements before and after the UV-C treatment.

The water activity of the ham was measured with an a_w_-Kryometer (AWK-40, NAGY Messsysteme GmbH, Gäufelden, Germany). Before each experiment the kryometer was calibrated with a 10% NaCl solution. The final a_w_-value for statistical analysis was the mean of three measurements.

For the determination of the moisture content, 3 g of the homogenized sample was mixed with sea sand and dried in a drying oven (Binder GmbH, Tuttlingen, Germany) at 103 °C for 4 h (ISO 1442:1997).

To analyze the ash concentration, 5 g of the homogenized sample were burned in a muffle furnace (Carbolite^®^, LAT GmbH, Garbsen, Germany) for 1–2 h at 600 °C (ISO 936:1998).

The protein concentration was calculated by analysis of nitrogen concentration, using the Kjeldahl method (Vapodest 50s^®^, Gerhardt Laboratory Systems GmbH, Koenigswinter, Germany) (ISO 937:1978).

Fat was determined after acid hydrolysis and extraction in Soxhlet equipment (LAT GmbH, Garbsen, Germany) (ISO 1443:1973).

The nitrite content was measured according to ISO 2916:1975. The sample was extracted with hot water and the proteins were precipitated. After filtration, sulfanilamide and naphthylethylenediamine hydrochloride were added. The presence of nitrite was shown by the formation of red complexes which was detected photometrically at 538 nm (Evolution 201-UV-VIS-Spectrophotometer, Thermo Scientific, Langenselbold, Germany).

The sodium chloride content was analyzed in accordance with ISO 1841:1996. The sample was extracted with hot water and clarified. The amount of chlorides was then measured by potentiometric titration using a 0.1 mol/L AgNO_3_ soluted in 0.3 mol/L HNO_3_.

To measure the antioxidant capacity, an ABTS [2,2′-azinobis-(3-ethyl-benzothiazoline-6-sulfonic acid)] radical cation solution was created as described by Re et al. [20]. ABTS was dissolved in water to a 7 mM concentration. To radicalize the solution 2.45 mM potassium persulfate was added. Before use, the ABTS^●^ radical solution was stored for 12–16 h in the dark at room temperature. The preparation of the samples and the measurement were performed according to Sacchetti et al. [21] with slight modifications. Approximately 1 g of the frozen meat sample added with 6 mL distilled water was transferred to a 50 mL tube and homogenized for 1 min at 30,000 rpm on ice with a Polytron PT 2500 homogenizer (Kinematica GmbH, Luzern, Switzerland). Afterwards, the tube was wrapped in an aluminum sheet and extracted in a shaker at 7 °C for 1 h. The homogenate was centrifuged for 15 min at 2340 *g* (Hermle Z383 K, Hermle Labortechnik, Wehingen, Germany). The bleaching rate of ABTS in the presence of the sample was monitored photometrically at 734 nm (Evolution 201-UV-VIS-Spectrophotometer, Thermo Scientific). To start the reaction, 2.50 mL ABTS^●^ radical solution was mixed with 20 µL supernatant of the centrifuged sample and 500 µL distilled water. The discoloration after 7 min was used to determine the antioxidant activity in µmol Trolox eq. per g of sample. For this, standard solutions with Trolox, the hydrophilic homologue of tocopherol, were prepared (0, 2.5, 5.0, 7.5, 10.0, 15.0 µM) and analyzed with the ABTS^●^ radical solution at 734 nm. For further analysis only calibration curves with correlation coefficients of at least 0.99 were used.

### 2.10. Statistical Analysis

The data of all experiments were statistically analyzed with SAS Enterprise Guide 7.1 (SAS Institute Inc., Cary, NC, USA) using the SAS PROC MIXED procedure considering the following model:

Y_ij_ = µ + D_i_ + B_j_ + ε_ij_ (2)

where Y_ij_ = observation value; µ = overall mean, D_i_ = fixed effect of UV-dose; B_j_ = random effect of batch; ε_ij_ = random error.

It was followed by the TUKEY multiple comparison test. All values were presented as means ± standard error (SE). Means were marked significant if the *p* value was lower than 0.05. All experiments were independently replicated at least three times.

## 3. Results and Discussion

### 3.1. Physicochemical and Microbial Quality of Ham before UV Treatment

The different quality parameters of ham as well as the nutritional information are shown in Table 1 and Table 2.

An a_w_-value of 0.93 ± 0.01, which was found in this study, is characteristic for raw cured ham [22,23]. The content of sodium nitrite was clearly below the maximum permitted limit of 150 mg/kg, specified in the Regulation (EC) No 1333/2008.

In comparison to the raw pork, the ham weighed 27.94% less. This was probably due to the moisture loss during the dry curing process. As a consequence, the relative proportion of the other components increased, as shown in previous studies [24,25]. A fresh pork LTL usually consists of up to 75.51% moisture, 21.79% protein, 2.02% fat, and 0.99% ash [26]. The cured ham of the present study instead only had 60.49% moisture, but 30.46% protein and 6.99% ash. The ash content also increased due to the addition of nitrite curing salt. Fat was removed before processing, so the proportion of fat in ham in this case was similar to raw pork.

The TVC with values between 1.89 and 2.07 log_10_ cfu/g ham (Table 2) was lower than stated in other publications about cured meat products [27,28] indicating good hygienic conditions during processing and storage of the ham. Fortunately, *Yersinia* spp., *Brochothrix* spp., and *Enterobacteriaceae* were not detected in the rolled fillets of ham neither on the treatment day (day 0) nor on the storage days 7 and 14 (Table 2).

### 3.2. Dose Dependent Bacterial Reductions on Ham

Table 3 presents that both species *Y. enterocolitica* and *B. thermosphacta* were significantly reduced after UV-C treatment. However, with an initial bacterial concentration of 10^8^ cfu/mL higher reductions of both bacterial species were detected (up to 1.36 and 1.52 log_10_ cfu/g for *Y*. *enterocolitica* and *B*. *thermosphacta*, respectively) in comparison to the ham inoculated with initial concentrations of 10^6^ cfu/mL (up to 1.03 and 0.94 log_10_ cfu/g for *Y*. *enterocolitica* and *B*. *thermosphacta*, respectively). In addition, there were no significant differences between both bacterial concentrations for the reductions on ham when the same UV-C dose was considered (Table 3). Therefore, an initial concentration of 10^8^ cfu/mL was chosen for further experiments.

Another study by Chun et al. [29] achieved higher reductions when irradiating sliced ham with doses of up to 800 mJ/cm^2^. In contrast to the present study, Chun et al. [29] found different UV-C sensitivities between various bacterial species. They detected the highest reductions for *Listeria* (*L.*) *monocytogenes* (by 2.74 log_10_ cfu/g), followed by *Salmonella* Typhimurium (2.02 log_10_ cfu/g) and *Campylobacter jejuni* (1.72 log_10_ cfu/g). Sommers et al. [30] reported a microbial reduction of 1.53–1.64 log_10_ cfu/g after UV-C treatments of Frankfurter sausages, but the authors detected no differences in results between different bacterial species analyzed (*L. monocytogenes*, *Staphylococcus aureus,* and *Salmonella*).

Isohanni and Lyhs [31] irradiated chicken meat with UV-C light and found that the reducing capacity of UV irradiation was better when using lower initial bacterial concentrations. In contrast, Butler et al. [32] demonstrated in in vitro experiments that resistance of bacteria to UV-C light is a cell characteristic and not concentration dependent. However, there are still other parameters that influence the effectiveness of UV-C light in decreasing bacterial counts such as the species, the strain, and the food composition [16]. Due to the porous surface of meat products and many influencing factors like protein or fat content, a reduction as high as e.g., on stainless steel surfaces or in in vitro experiments cannot be expected. With a UV-C dose of 400 mJ/cm^2^ Sommers et al. [30] reduced pathogens by >5 log_10_ cfu/g on stainless steel. Kim et al. [33] demonstrated a reduction of at least 5 log_10_ cfu/g when irradiating a bacterial suspension with 30–60 mJ/cm^2^. Hence, even with lower doses a higher decrease of bacteria was seen, but the results cannot be transferred to an application on meat or meat products. This might indicate that various parameters have to be taken into consideration when irradiation meat and meat products, but the surface texture is certainly the most influencing factor.

The present study has shown that UV-C light can reduce microorganisms on RTE meat products. It should be noted that in vitro experiments have shown that the reduction effect might be species- or strain-dependent [34]. However, as already described, the bacterial strains were chosen based on previous in vitro experiments and their porcine origin. In addition, the study like other studies before it [29,35], did not aim at demonstrating individual strain-specific effects.

### 3.3. Antimicrobial Effect of UV-C Irradiation on Ham during Storage

The decontamination efficacy of UV-C treatments against *Y. enterocolitica* and *B. thermosphacta* on ham during refrigerated storage is presented in Table 4. On all days, significantly lower concentrations of both bacterial species after UV-C treatments in comparison to the untreated control samples were seen. No significant differences of the reductions were obtained between the two different UV-C doses for inoculated *B. thermosphacta* and *Y. enterocolitica,* except for the *Yersinia* on day 0. On this day the higher dose of 4080 mJ/cm^2^ resulted in significantly lower *Yersinia* counts on the ham compared to the 408 mJ/cm^2^ treated ham samples.

The presented results regarding the reduction rates, mainly agree with other studies. For example, Chun et al. [14] achieved maximum reductions of up to 1.29 log_10_ cfu/g after irradiating chicken breast with 500 mJ/cm^2^ and storage for 6 days. Similar results were presented by Lyon et al. [36] who treated broiler breast fillets with UV-C doses of 300 mJ/cm^2^, whereas Lázaro et al. [15] showed slightly lower reductions of up to 0.6 log_10_ cfu/g during refrigerated storage of chicken meat irradiated with an UV-C of dose of 175 mJ/cm^2^.

In contrast to the present study, Chun et al. [29] eliminated *L. monocytogenes* by up to 2.7 log_10_ cfu/g on sliced ham with doses ranging from 100 to 800 mJ/cm^2^. They showed that the reduction rates remained stable during storage up to 9 days. However, since the efficiency of UV-C is also dependent on the used UV source as well as on the experimental design, it can only be assumed that the different surface topology of ham in comparison to meat has made a difference in the reduction rates of microorganisms. In experiments from our group using similar experimental conditions, comparable reductions of up to 0.9 log_10_ cfu/g were found for *Y. enterocolitica* and *B. thermosphacta* on pork irradiated with 408 mJ/cm^2^ [37]. These data indicated that there is no difference in effects of UV-C treatments on meat or meat products, despite the higher salt and nitrite content and the lower a_w_ values in ham. However, it must be considered that both meat and ham have a porous and uneven surface, which might absorb or attenuate UV-C light before it can reach the bacteria.

Although microbial contamination often occurs on the surface of the product only, bacteria might migrate deeper into the tissue during storage, which protects them from surface decontamination methods like UV-C light [38]. This migration might have occurred also in the present study, since higher UV doses did not further reduce the bacterial loads, despite a significant initial reduction of bacteria. Furthermore, we have demonstrated a very high initial bacterial load of ham, so it is of course possible that other reduction values are achieved with a lower initial bacterial load.

Gardner and Shama [39] suggested that polymeric substances produced by microorganisms used to adhere to surfaces might attenuate UV-C light. This could be another reason why no higher reductions were achieved. Nevertheless, the current results showed that the antibacterial effects of UV-C treatment despite quite small reductions can contribute to increased safety of rolled fillets of ham during storage thereby helping to counteract possible bacterial contaminations, which may occur during the production process. In this context it should be considered that the application of different preservation methods (“hurdle principle”) is always useful to increase food safety without negative effects on other quality parameters like color.

### 3.4. Photoreactivation on Ham

**Figure 2:**
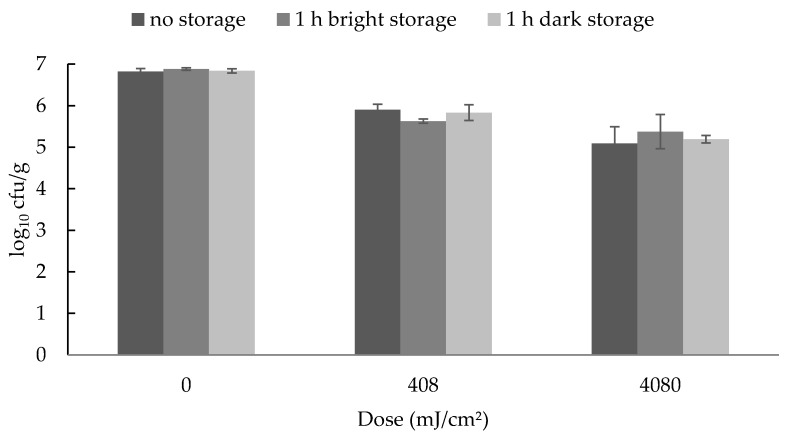
Mean and standard error values of the bacterial counts of *Yersinia enterocolitica* inoculated on ham immediately after UV-C treatment and after 1 h of light or dark storage (*n* = 3). Since no statistically significant differences occurred, no letters are given.

**Figure 3:**
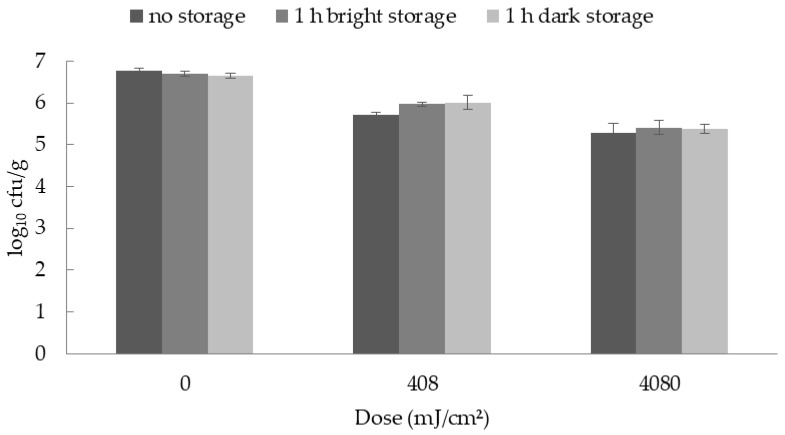
Mean and standard error values of the bacterial counts of *Brochothrix thermosphacta* inoculated on ham immediately after UV-C treatment and after 1 h of light or dark storage (*n* = 3). Since no statistically significant differences occurred, no letters are given.

The subsequent exposure of the inoculated and irradiated ham to light had no effect on the bacterial reductions. There were no significant differences between the different treatment groups (Figure 2 and Figure 3).

In in vitro experiments, it could be shown that photoreactivation occurs and bacterial counts increase when irradiated samples are exposed to visible light for some time. For example, Sommer et al. [35] found that direct plating of UV-irradiated suspensions resulted in a reduction of up to 6 log_10_, however, 30 and 60 min of illumination led to an increase of the microorganisms of 1 and 3 log_10_.

Bacteria must be exposed to visible light within a few hours after the UV-C irradiation process to allow photoreactivation to take place. Otherwise, the damage cannot be repaired anymore [40]. As in the food production the products are stored under dark conditions after processing, photoreactivation might not be initiated.

Furthermore, Sanz et al. [41] found out that photoreactivation does not occur if very high UV-C doses are used, because in this case the DNA damage is too high. In in vitro studies, lower UV-C doses could be used because radiation can penetrate watery suspensions more easily. For meat and meat products higher doses are required because of the solid and inhomogeneous structure of the surface. Therefore, the bacteria that are affected by those high doses of UV-C light are not able to survive due to the irreversible damage. Other parameters have to be taken into consideration that prevent the reactivation of bacteria after UV-C treatment as the growing conditions on cured ham are not optimal for *Y. enterocolitica* and *B. thermosphacta*. However, other studies have shown that factors such as pH- and a_w_-value did not affect the resistance or sensitivity of bacteria to UV-C light [42,43] and therefore might not play a major role in the photoreactivation of bacteria on ham.

### 3.5. Effects of UV-C Light on the Color Values and Antioxidant Capacity of Ham

Due to photochemical reactions and the formation of free radicals, UV-C light may lead to oxidation processes, degradation of antioxidants, and color changes [44]. Therefore, the meat color and the antioxidant capacities of the ham were also analyzed (Table 5, Table 6 and Table 7).

On day 0 no significant changes of the color values between irradiated and non-irradiated samples could be found. On days 7 and 14 the a* values of the samples irradiated with a dose of 4080 mJ/cm^2^ were significantly lower and the b* value significantly higher compared to the untreated and treated (408 mJ/cm^2^) ham. Additionally, the color difference ΔE was calculated (Table 6). If ΔE is smaller than 3.5, no color difference is noticed by the consumer [45]. Only on day 0, ΔE values above 3.5 for the higher UV-C doses comparing to the untreated samples could be determined, whereas all other results were below 3.5. Comparing the ΔE values between the UV-C doses the results on days 7 and 14 are significantly different. This shows that higher UV-C doses lead to greater color changes.

As far as we know, no studies have been published that analyzed the effects of UV-C treatments on the color of meat products like ham. Therefore, studies that investigated UV-C treatment of meat are considered in the following discussion, although the rolled fillets of ham were more stable in color than raw meat due to the addition of sodium nitrite. Lyon et al. [37] reported lower a* and higher b* values of irradiated chicken breast fillets on day 7 of storage after treatment with a clearly lower dose of 300 mJ/cm^2^. Lázaro et al. [15] observed lower L* values of UV-C treated chicken meat during refrigerated storage in comparison to untreated samples, whereas Wallner-Pendleton et al. [46] found no significant color changes when irradiating chicken breast. In experiments from our laboratory, no significant effect of UV-C treatments with 408 and 2040 mJ/cm^2^ on the color values of pork was found on days 0, 7, and 14 of storage [38]. Due to the inhomogeneity of the different results, it remains unclear which color values are affected by UV-C light and at which certain UV-C dose those color changes may occur when considering the L*, a*, and b* values. The significant differences of the ΔE values between the UV-C doses chosen indicate that higher doses lead to greater color changes. Although the color difference (ΔE) results, except for the day 0 results using 4080 mJ/cm^2^, are probably not visible for the consumers considering Higuero et al. [45], for practical application UV-C doses must be chosen carefully to avoid an influence of the consumer, especially of visually more sensitive persons.

Lipid oxidation can alter the organoleptic properties of food. It might induce off-flavors and color changes and subsequently reduce the consumer’s acceptance [47]. Oxidation processes can be decreased or avoided if antioxidants were used to counteract them. As these antioxidant contents might be reduced by UV-C treatment, antioxidant capacities were analyzed using the ABTS method to give an insight into possible oxidative antioxidative interactions. In the present study, a UV-C treatment with 4080 mJ/cm^2^ on day 0 resulted in an increase of the antioxidant capacity in comparison to the untreated and treated (408 mJ/cm^2^) ham samples. However, on days 7 and 14, no significant difference of the antioxidant capacities could be seen. In general, a higher antioxidant capacity is beneficial for the product, but it is suggested not to overestimate the result on day 0. A higher antioxidant capacity did not occur during further storage and was probably not a result of the UV-C treatment.

Cichoski et al. [48] investigated the effect of UV-C light on oxidative processes of chicken drumsticks irradiated with doses of 540 and 946 mJ/cm^2^ and showed that an UV-C irradiation did not lead to lipid or protein oxidation. Lázaro et al. [15] analyzed chicken meat irradiated with up to 175.5 mJ/cm^2^ and also found that it did not promote oxidation processes. Both studies support the results of the present study. However, it cannot be excluded that higher UV-C doses may induce oxidation processes and that raw and processed meat react differently to the treatment as the results of the present study can of course only give a first indication of the antioxidant activity and of oxidative changes. Therefore, further studies are necessary, e.g., evaluating the lipid oxidation or protein oxidation, to allow a more valid statement about the influence of UV-C irradiation.

## 4. Conclusions

The present study shows that UV-C irradiation reduces the microbial load on ham without causing major quality changes. Although color changes were detected on storage days 7 and 14, they can be regarded as minor and may not influence the consumer’s acceptance of the product. A significant reduction of both *Y. enterocolitica* and *B. thermosphacta* was seen on all storage days in comparison to the non-irradiated samples. Although the reduction of the bacteria counts in the present study was quite small, it has to be kept in mind that in a food company every reduction of bacteria contaminations contributes to better product hygiene and that probably more than one preservation method needs to be applied (“hurdle principle”) to get a safe product. The method might be more beneficial in combination with other decontamination technologies, which not only affect the product surface but also deeper layers, so that more bacteria may be eliminated.

## Figures and Tables

**Table 1:** Mean and standard error values of different quality parameters of the rolled fillets of ham after 6 weeks of storage/ripening (*n* = 3).

Parameter	Ham
L*	44.1 ± 1.0
a*	7.3 ± 0.3
b*	3.9 ± 0.1
a_w_-value	0.93 ± 0.01
Moisture (%)	60.49 ± 0.31
Ash (%)	6.99 ± 0.25
Protein (%)	30.46 ± 0.24
Fat (%)	2.01 ± 0.48
NaNO_2_ (mg/kg)	8.56 ± 1.09
NaCl (g/kg)	58.0 ± 2.8

**Table 2:** Mean and standard error values of microbiological parameters of the rolled fillets of ham on the treatment day and storage days 7 and 14 (*n* = 3).

Parameter	Day 0	Day 7	Day 14
Total viable count	1.89 ± 0.15	1.81 ± 0.05	2.07 ± 0.05
*Enterobacteriaceae*	0.7 ± 0.0	0.7 ± 0.0	0.7 ± 0.0
*Yersinia* spp.	1.7 ± 0.0	1.7 ± 0.0	1.7 ± 0.0
*Brochothrix* spp.	1.7 ± 0.0	1.7 ± 0.0	1.7 ± 0.0

All values in log_10_ colony forming units (cfu)/g, detection limits for quantification were 1.0 log_10_ cfu/g (total viable count and *Enterobacteriaceae*) or 2.0 log_10_ cfu/g (*Yersinia* spp. and *Brochothrix* spp.); if no colonies were determined on the agar plates with the initial dilution, the half detection limit (0.7 log_10_ or 1.7 log_10_ cfu/g meat) was used for further calculations.

**Table 3:** Mean and standard error values of the bacterial counts of *Yersinia enterocolitica* (*n* = 5) and *Brochothrix thermosphacta* (*n* = 3) on ham after ultraviolet (UV)-C irradiation depending on the initial bacterial concentration.

	*Yersinia enterocolitica*		*Brochothrix thermosphacta*	
UV-C Dose (mJ/cm^2^)	Inital Concentration Used		Inital Concentration Used	
	10^8^ cfu/mL	10^6^ cfu/mL	10^8^ cfu/mL	10^6^ cfu/mL
0	6.76 ^a^ ± 0.07	4.94 ^a^ ± 0.03	6.40 ^a^ ± 0.11	4.80 ^a^ ± 0.05
408	6.06 ^ab^ ± 0.12	4.14 ^ab^ ± 0.17	5.62 ^ab^ ± 0.20	4.10 ^b^ ± 0.15
2040	5.79 ^b^ ± 0.25	3.91 ^b^ ± 0.34	5.40 ^ab^ ± 0.20	3.86 ^b^ ± 0.24
4080	5.70 ^b^ ± 0.33	3.93 ^b^ ± 0.40	5.00 ^b^ ± 0.41	4.01 ^b^ ± 0.13
6120	5.40 ^b^ ± 0.32	4.24 ^ab^ ± 0.14	4.88 ^b^ ± 0.54	3.96 ^b^ ± 0.18

All values in log_10_ colony forming units/g. ^a, b^ Values in a column within the same group followed by a different letter differ significantly (*p* ≤ 0.05).

**Table 4:** Mean and standard error values of the bacterial counts of *Yersinia enterocolitica* and *Brochothrix thermosphacta* on rolled fillets of ham depending on the UV-C dose and storage day (*n* = 3).

Dose (mJ/cm^2^)	Day 0		Day 7		Day 14	
	Yersinia	Brochothrix	Yersinia	Brochothrix	Yersinia	Brochothrix
0	7.03 ^a^ ± 0.03	6.11 ^a^ ± 0.07	6.99 ^a^ ± 0.06	6.01 ^a^ ± 0.05	6.58 ^a^ ± 0.04	5.72 ^a^ ± 0.20
408	6.40 ^b^ ± 0.07	5.61 ^b^ ± 0.09	6.17 ^b^ ± 0.11	5.47 ^b^ ± 0.01	6.13 ^b^ ± 0.09	5.06 ^b^ ± 0.03
4080	5.92 ^c^ ± 0.13	5.46 ^b^ ± 0.06	6.12 ^b^ ± 0.12	5.49 ^b^ ± 0.05	5.84 ^b^ ± 0.09	4.93 ^b^ ± 0.08

All values in log_10_ colony forming units/g. ^a, b, c^ Values in a column within the same group followed by a different letter differ significantly (*p* ≤ 0.05).

**Table 5:** Mean and standard error values of the color results of rolled fillets of ham depending on the UV-C dose and storage day (*n* = 3).

Dose (mJ/cm^2^)	Day 0			Day 7			Day 14		
	L*	a*	b*	L*	a*	b*	L*	a*	b*
0	48.9 ± 0.7	10.5 ± 1.0	9.4 ± 1.0	48.8 ± 0.8	10.3 ^a^ ± 0.5	8.8 ^a^ ± 0.5	48.3 ± 1.1	10.4 ^a^ ± 0.2	8.5 ^a^ ± 0.2
408	48.4 ± 0.9	8.3 ± 0.9	9.2 ± 1.1	47.8 ± 0.7	10.6 ^a^ ± 0.5	9.0 ^a^ ± 0.3	48.0 ± 0.8	10.6 ^a^ ± 0.3	8.9 ^a^ ± 0.2
4080	49.1 ± 0.4	7.3 ± 1.3	10.1 ± 0.8	48.3 ± 1.2	9.0 ^b^ ± 0.2	10.2 ^b^ ± 0.5	48.1 ± 1.0	9.5 ^b^ ± 0.2	10.3 ^b^ ± 0.3

^a, b^ Values in a column within the same group followed by a different letter differ significantly (*p* ≤ 0.05).

**Table 6:** Mean and standard error values for the color differences (ΔE) between the untreated and UV-C irradiated samples (*n* = 3).

	Difference between 0 and 408 mJ/cm^2^	Difference between 0 and 4080 mJ/cm^2^
Day 0	2.63 ± 1.46	4.03 ± 0.31
Day 7	1.13 ^y^ ± 0.33	2.21 ^x^ ± 0.08
Day 14	0.71 ^y^ ± 0.03	2.14 ^x^ ± 0.06

^x, y^ Values in a line at the same day followed by a different letter differ significantly (*p* ≤ 0.05).

**Table 7:** Mean and standard error values of the antioxidant capacities of rolled fillets of ham depending on the UV-C dose and storage day (*n* = 3).

Dose (mJ/cm^2^)	Day 0	Day 7	Day 14
0	3.2 ^a^ ± 0.2	3.2 ± 0.2	3.3 ± 0.2
408	3.4 ^a^ ± 0.2	3.4 ± 0.3	3.3 ± 0.1
4080	3.6 ^b^ ± 0.2	3.3 ± 0.2	3.1 ± 0.2

All values in µmol Trolox eq./g. ^a, b^ Values in a column within the same group followed by a different letter differ significantly (*p* ≤ 0.05).

## References

[1] Sommers C.H., Boyd G. (2006). Variations in the radiation sensitivity of foodborne pathogens associated with complex ready-to-eat food products. Radiat. Phys. Chem..

[2] Fredriksson-Ahomaa M., Koch U., Klemm C., Bucher M., Stolle A. (2004). Different genotypes of *Yersinia enterocolitica* 4/O:3 strains widely distributed in butcher shops in the Munich area. Int. J. Food Microbiol..

[3] Mataragas M., Skandamis P.N., Drosinos E.H. (2008). Risk profiles of pork and poultry meat and risk ratings of various pathogen/product combinations. Int. J. Food Microbiol..

[4] Grahek-Ogden D., Schimmer B., Cudjoe K.S., Nygård K., Kapperud G. (2007). Outbreak of *Yersinia enterocolitica* Serogroup O:9 Infection and Processed Pork, Norway. Emerg. Infect. Dis..

[5] European Centre for Disease Prevention and Control (ECDC) (2018). Yersiniosis. Annual Epidemiological Reports.

[6] European Food Safety Authority (EFSA) (2007). Monitoring and identification of human enteropathogenic *Yersinia* spp.—Scientific Opinion of the Panel on Biological Hazards. EFSA J..

[7] Råsbäck T., Rosendal T., Stampe M., Sannö A., Aspán A., Järnevi K., Lahti E.T. (2018). Prevalence of human pathogenic *Yersinia enterocolitica* in Swedish pig farms. Acta Vet. Scand..

[8] Hultman J., Rahkila R., Ali J., Rousu J., Björkroth K.J. (2015). Meat Processing Plant Microbiome and Contamination Patterns of Cold-Tolerant Bacteria Causing Food Safety and Spoilage Risks in the Manufacture of Vacuum-Packaged Cooked Sausages. Appl. Environ. Microbiol..

[9] Jayasena D.D., Jo C. (2013). Essential oils as potential antimicrobial agents in meat and meat products: A review. Trends Food Sci. Technol..

[10] Bhaduri S., Smith J.L., Labbé R.G., Garcia S. (2013). Yersinia enterocolitica. Guide to Foodborne Pathogens.

[11] Stanborough T., Fegan N., Powell S.M., Tamplin M., Chandry P.S. (2017). Insight into the Genome of *Brochothrix thermosphacta*, a Problematic Meat Spoilage Bacterium. Appl. Environ. Microbiol..

[12] Gayán E., Condón S., Álvarez I. (2014). Biological Aspects in Food Preservation by Ultraviolet Light: A Review. Food Bioprocess Technol..

[13] Goosen N., Moolenaar G.F. (2008). Repair of UV damage in bacteria. DNA Repair.

[14] Chun H.H., Kim J.Y., Lee B.D., Yu D.J., Song K.B. (2010). Effect of UV-C irradiation on the inactivation of inoculated pathogens and quality of chicken breasts during storage. Food Control.

[15] Lázaro C.A., Conte-Júnior C.A., Monteiro M.L.G., Canto A.C.V.S., Costa-Lima B.R.C., Mano S.B., Franco R.M. (2014). Effects of ultraviolet light on biogenic amines and other quality indicators of chicken meat during refrigerated storage. Poult. Sci..

[16] Guerrero-Beltrán J.A., Barbosa-Cánovas G.V. (2004). Review: Advantages and Limitations on Processing Foods by UV Light. Food Sci. Technol. Int..

[17] Sastry S.K., Datta A.K., Worobo R.W. (2000). Ultraviolet Light. J. Food Sci..

[18] Kamat A.S., Khare S., Doctor T., Nair K.M. (1997). Control of *Yersinia enterocolitica* in raw pork and pork products by γ-irradiation. Int. J. Food Microbiol..

[19] Hechelmann H. (1981). Vorkommen und Bedeutung von Brochothrix thermosphacta bei Kühllagerung von Fleisch und Fleischerzeugnissen.

[20] Re R., Pellegrini N., Proteggente A., Pannala A., Yang M., Rice-Evans C. (1999). Antioxidant activity applying an improved ABTS radical cation decolorization assay. Free Radic. Biol. Med..

[21] Sacchetti G., Mattia C.D., Pittia P., Martino G. (2008). Application of a radical scavenging activity test to measure the total antioxidant activity of poultry meat. Meat Sci..

[22] Krämer J., Prange A. (2016). Lebensmittel-Mikrobiologie.

[23] Sinell H.-J. (2004). Einführung in die Lebensmittelhygiene.

[24] Gil M., Guerrero L., Sárraga C. (1999). The effect of meat quality, salt and ageing time on biochemical parameters of dry-cured *Longissimus dorsi* muscle. Meat Sci..

[25] Seong P.N., Park K.M., Kang S.M., Kang G.H., Cho S.H., Park B.Y., Ba H.V. (2014). Effect of Particular Breed on the Chemical Composition, Texture, Color, and Sensorial Characteristics of Dry-cured Ham. Asian-Aust. J. Anim. Sci..

[26] Kim J.H., Seong P.N., Cho S.H., Park B.Y., Hah K.H., Yu L.H., Lim D.G., Hwang I.H., Kim D.H., Lee J.M. (2008). Characterization of Nutritional Value for Twenty-one Pork Muscles. Asian-Aust. J. Anim. Sci..

[27] Aliño M., Grau R., Toldrá F., Blesa E., Pagán M.J., Barat J.M. (2010). Physicochemical properties and microbiology of dry-cured loins obtained by partial sodium replacement with potassium, calcium and magnesium. Meat Sci..

[28] Vilar I., Fontán M.C.G., Prieto B., Tornadijo M.E., Carballo J. (2000). A survey on the microbiological changes during the manufacture of dry-cured lacón, a Spanish traditional meat product. J. Appl. Microbiol..

[29] Chun H., Kim J., Chung K., Won M., Song K.B. (2009). Inactivation kinetics of *Listeria monocytogenes*, *Salmonella enterica* serovar Typhimurium, and *Campylobacter jejuni* in ready-to-eat sliced ham using UV-C irradiation. Meat Sci..

[30] Sommers C.H., Sites J.E., Musgrove M. (2010). Ultraviolet Light (254 nm) inactivation of pathogens on foods and stainless steel surfaces. J. Food Saf..

[31] Isohanni P.M.I., Lyhs U. (2009). Use of ultraviolet irradiation to reduce *Campylobacter jejuni* on broiler meat. Poult. Sci..

[32] Butler R.C., Lund V., Carlson D.A. (1987). Susceptibility of Cambylobacter jejuni and Yersinia enterocolitica to UV Radiation. Appl. Environ. Microbiol..

[33] Kim T., Silva J.L., Chen T.C. (2002). Effects of UV Irradiation on Selected Pathogens in Peptone Water and on Stainless Steel and Chicken Meat. J. Food Prot..

[34] Sommer R., Lhotsky M., Haider T., Cabaj A. (2000). UV Inactivation, Liquid-Holding Recovery, and Photoreactivation of *Escherichia coli* O157 and Other Pathogenic *Escherichia coli* Strains in Water. J. Food Prot..

[35] Yeh Y., de Moura F.H., van den Broek K., de Mello A.S. (2018). Effect of ultraviolet light, organic acids, and bacteriophage on *Salmonella* populations in ground beef. Meat Sci..

[36] Lyon S.A., Fletcher D.L., Berrang M.E. (2007). Germicidal Ultraviolet Light to Lower Numbers of *Listeria monocytogenes* on Broiler Breast Fillets. Poult. Sci..

[37] Reichel J., Kehrenberg C., Krischek C. (2019). Inactivation of *Yersinia enterocolitica* and *Brochothrix thermosphacta* on pork by UV-C irradiation. Meat Sci..

[38] Richards G.M., Beuchat L.R. (2005). Infection of cantaloupe rind with *Cladosporium cladosporioides* and *Penicillium expansum*, and associated migration of *Salmonella poona* into edible tissues. Int. J. Food Microbiol..

[39] Gardner D.W.M., Shama G. (2000). Modeling UV-Induced Inactivation of Microorganisms on Surfaces. J. Food Prot..

[40] Hallmich C., Gehr R. (2010). Effect of pre- and post-UV disinfection conditions on photoreactivation of fecal coliforms in wastewater effluents. Water Res..

[41] Sanz E.N., Dávila I.S., Balao J.A.A., Alonso J.M.Q. (2007). Modelling of reactivation after UV disinfection: Effect of UV-C dose on subsequent photoreactivation and dark repair. Water Res..

[42] Gayán E., García-Gonzalo D., Álvarez I., Condón S. (2014). Resistance of *Staphylococcus aureus* to UV-C light and combined UV-heat treatments at mild temperatures. Int. J. Food Microbiol..

[43] Gayán E., Serrano M.J., Pagán R., Álvarez I., Condón S. (2015). Environmental and biological factors influencing the UV-C resistance of *Listeria monocytogenes*. Food Microbiol..

[44] Falguera V., Pagán J., Garza S., Garvín A., Ibarz A. (2011). Ultraviolet processing of liquid food: A review—Part 2: Effects on microorganisms and on food components and properties. Food Res. Int..

[45] Higuero N., Moreno I., Lavado M.C., Vidal-Aragon M.C., Cava R. (2020). Reduction of nitrate and nitrite in Iberian dry cured loins and its effects during drying process. Meat Sci..

[46] Wallner-Pendleton E.A., Sumner S.S., Froning G.W., Stetson L.E. (1994). The Use of Ultraviolet Radiation to Reduce *Salmonella* and Psychrotrophic Bacterial Contamination on Poultry Carcasses. Poult. Sci..

[47] Park S.Y., Chin K.B. (2011). Antioxidant activities of pepsin hydrolysates of water- and salt-soluble protein extracted from pork hams. Int. J. Food Sci. Technol..

[48] Cichoski A.J., Moura H.C., Silva M.S., Rampelotto C., Wagner R., Barin J.S., Vendruscolo R.G., Dugatto J.S., Athayde D.R., Costa M.A.D. (2015). Oxidative and microbiological profiles of chicken drumsticks treated with ultraviolet-C radiation. J. Food Process. Preserv..

